# Case report: ACTH-secreting pituitary carcinoma metastatic to the liver in a patient with a history of atypical pituitary adenoma and Cushing’s disease

**DOI:** 10.1186/s13000-017-0624-5

**Published:** 2017-04-18

**Authors:** Amy S. Joehlin-Price, Douglas A. Hardesty, Christina A. Arnold, Lawrence S. Kirschner, Daniel M. Prevedello, Norman L. Lehman

**Affiliations:** 1 Department of Pathology, The Ohio State University Wexner Medical Center, 410 W 10th Avenue, Columbus, OH 43210 USA; 2 Department of Neurosurgery, The Ohio State University Wexner Medical Center, 410 W 10th Avenue, Columbus, OH 43210 USA; 3 Department of Internal Medicine, The Ohio State University Wexner Medical Center, 410 W 10th Avenue, Columbus, OH 43210 USA

**Keywords:** Case report, Pituitary carcinoma, ACTH, Liver metastasis, Atypical pituitary adenoma, Cushing’s disease

## Abstract

**Background:**

Pituitary carcinoma is a rare entity requiring the presence of metastasis to confirm its malignant potential. We report a case of pituitary carcinoma and discuss the diagnosis and management of this lesion in relation to the existing literature.

**Case presentation:**

The patient is a 51-year-old woman with Cushing’s disease and intact adrenal glands who was diagnosed with metastatic pituitary carcinoma to the liver, 29 months after initial resection of an ACTH-secreting primary atypical pituitary adenoma (APA). Prior to detection of this metastasis the patient underwent repeat resection and radiotherapy for residual cavernous sinus disease. The metastatic lesion was detected by interval surveillance of serum ACTH and 24-hour urine cortisol, which despite stable pituitary MRI, were significantly elevated. These abnormalities prompted a PET scan that demonstrated hypermetabolic liver parenchyma, which was suspicious for metastasis on abdominal MRI. An ultrasound-guided liver biopsy demonstrated nests of moderately-differentiated cells with intermediate-sized, monotonous nuclei, distinct nucleoli, and abundant basophilic cytoplasm, confirmed by immunohistochemistry to represent metastatic pituitary carcinoma. The liver lesion was subsequently successfully removed by wedge resection. One year later, the patient’s residual cavernous sinus disease grew markedly, and she was placed on dual-agent chemotherapy consisting of oral temozolomide and capecitabine, with stabilization of her intracranial disease to present, although liver metastases recurred.

**Conclusions:**

Pituitary carcinoma is a rare entity impossible to recognize as a primary tumor because its diagnosis by definition requires the presence of metastasis. Maintaining awareness of the entity and its precursor lesion APA is essential for its accurate pathologic diagnosis and appropriate management.

A version of this abstract has been previously published and presented at the 2015 Society of Neuro-Oncology Annual Meeting [1].

## Background

Pituitary carcinoma is a rare entity, accounting for only 0.1 to 0.2% of pituitary tumors [2–4], and for which there are few effective treatments. Pituitary carcinoma cannot be diagnosed by pituitary imaging or biopsy alone, as cerebrospinal or systemic metastasis is required for its diagnosis. This makes early clinical detection extremely challenging.

Most pituitary carcinomas are functional in nature, predominantly secreting prolactin or adrenocorticotropic hormone (ACTH) [3, 5]. Lesions secreting growth hormone [5], both follicle stimulating hormone (FSH) and luteinizing hormone (LH) [6], and thyroid stimulating hormone (TSH) [5] are less commonly reported, as are non-secreting carcinomas [7–9]. Initially, corticotroph carcinomas were thought to be generally associated with Nelson’s syndrome [3, 10, 11], but Cushing’s-associated cases have also been reported [12–19]. Herein, we present an additional rare case of an ACTH-secreting atypical pituitary adenoma (APA) previously treated with two resections and radiation therapy, metastasizing to the liver in a female patient with Cushing’s disease. We discuss the clinical and pathologic diagnosis and management of this patient and briefly review the pertinent literature.

## Case presentation

A now 52-year-old woman with a history of type 2 diabetes mellitus well-controlled by metformin initially presented at age 49 to another institution with complaints of blurry vision. Work-up revealed a pituitary macroadenoma, invasive into the left cavernous sinus as assessed by magnetic resonance imaging (MRI). The referring neurosurgery service performed a partial resection of the lesion by an endoscopic endonasal approach, resulting in symptomatic improvement for the patient. Review of this original pathology showed a population of fairly monotonous polygonal cells with coarse chromatin, distinct nucleoli, and small amounts of granular, basophilic cytoplasm. Immunohistochemical staining for chromogranin A and ACTH were positive, confirming the lesion as a functional pituitary tumor. A diagnosis of atypical pituitary macroadenoma was rendered due to positive p53 staining and an elevated Ki-67 proliferation index of 4%.

**Fig. 1:**
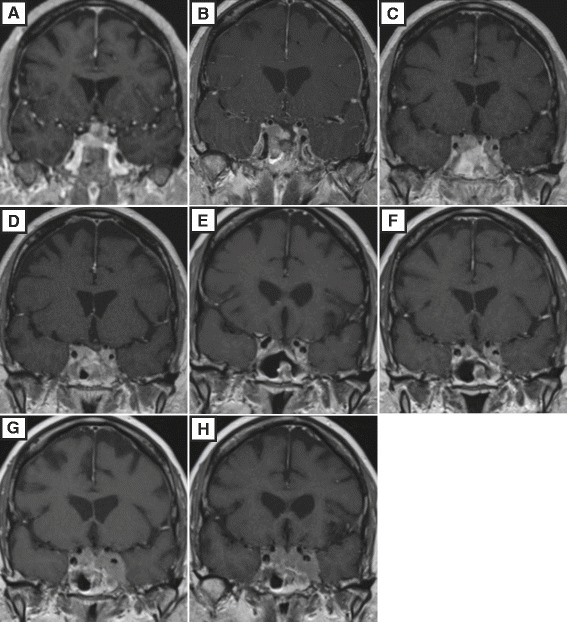
MRI of the pituitary throughout the patient’s clinical course. Coronal T1-weighted post-gadolinium images are shown. After partial resection at another facility, she presented to us for more definitive endoscopic endonasal resection of the residual sellar tumor (**a**), which was successful (**b**). She then underwent radiation therapy 3 months after this repeat surgery, at which point her cavernous sinus residual disease had already subtly progressed (**c**). Radiation therapy was initially successful for local control of disease 3 months after completion of fractionated IMRT (**d**). Two months following this MRI, her ACTH levels increased dramatically despite stability of her cavernous sinus residual disease (**e**), and metastasis to the liver was diagnosed (see Fig. 4). Six months after resection of the liver metastasis, her sellar and cavernous sinus disease remained stable (**f**), but then quickly progressed over the next 6 months (**g**). She was started on dual-agent oral chemotherapy, and 8 months later her local disease was stable (**h**), although she has suffered additional liver metastases

The patient’s symptoms including blurry vision and headaches gradually recurred. She presented to our institution 8 months later for a second opinion and repeat resection. At this point, an MRI demonstrated a recurrent pituitary lesion occupying the sellar and suprasellar regions with mass effect on the optic chiasm and extension into the left cavernous sinus (Fig. 1a). On the day of surgery, ACTH measured 259 pg/mL (normal range 9–50 pg/mL). An endoscopic endonasal transplanum surgical approach was performed and all of the sellar and suprasellar components of the recurrent tumor were resected (Fig. 1b). The tumor was fibrous and adherent to the optic apparatus, requiring lengthy sharp dissection. Intraoperative assessment concluded that microscopic residual tumor remained in the walls of the bilateral cavernous sinuses.

**Fig. 2:**
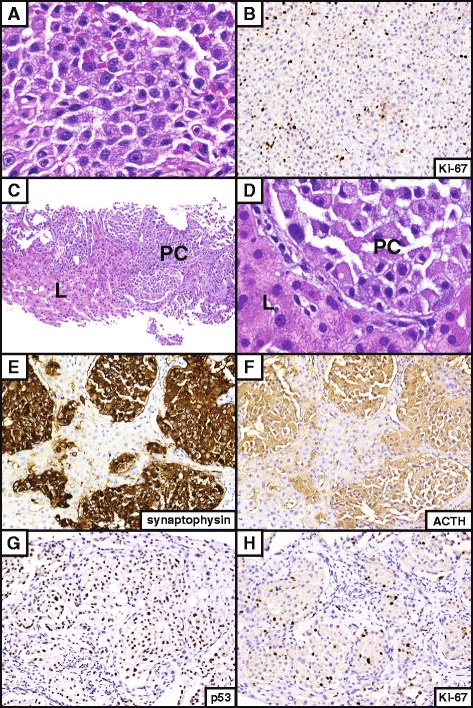
Histology of the APA and pituitary carcinoma. Hematoxylin and eosin (H&E) stain of the recurrent sellar-region APA demonstrating mildly pleomorphic polygonal cells with distinct nucleoli (**a**). Immunohistochemical staining for Ki-67 yielded a proliferation index of 8 to 15% (**b**). H&E stained views of the metastatic pituitary carcinoma, with the carcinoma labeled “PC” and surrounding benign liver labeled “L” for reference (**c**, **d**). Note the similar histologic appearance to the recurrent sellar lesion above. Mitotic activity was not conspicuous and necrosis was not present in any of the three lesions. Immunostains for synaptophysin (**e**), ACTH (**f**) and p53 (**g**) all demonstrated diffuse immunoreactivity in the metastatic lesional cells. Ki-67 was positive in approximately 10% of tumor nuclei overall, with focal areas approaching 20% immunostaining (**h**). Original magnifications: **a**, 600×; **b**, 200×; **c**, 100×; **d**, 600×; and **e**–**h**, 200x

Pathologic examination revealed similar morphologic findings to the previous specimen, however the Ki-67 labeling index had increased to approximately 8% overall and was focally up to 15% (Fig. 2a & b). A diagnosis of recurrent atypical pituitary adenoma was thus rendered. Next, intensity modulated radiation therapy (IMRT) was administered at a dose of 5040 cGY in 28 fractions. Slight growth of the cavernous sinus tumor was noted even prior to initiation of radiation therapy (Fig. 1c). She tolerated surgery and radiotherapy well with relief from her neurologic complaints and stabilization of the lesions after several months (Fig. 1d). She was started on pasireotide after completion of IMRT but could not tolerate the medication long-term due to hyperglycemia.

**Fig. 3:**
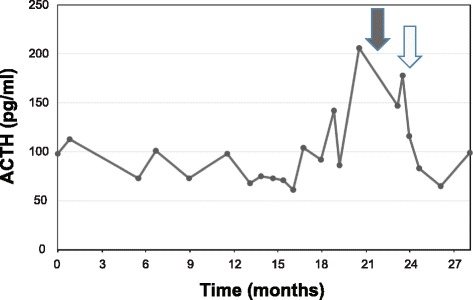
Serum ACTH levels during the patient’s clinical course. Twenty-one months after the second pituitary tumor resection, there was a marked elevation of serum ACTH, despite stability of her sellar disease. This occurred immediately prior to discovery of her liver lesion (*dark arrow*). The serum ACTH abruptly declined after resection of the metastasis (*light arrow*). A similar decline in systemic ACTH has been previously reported following resection of a corticotroph carcinoma liver metastasis (19)

**Fig. 4:**
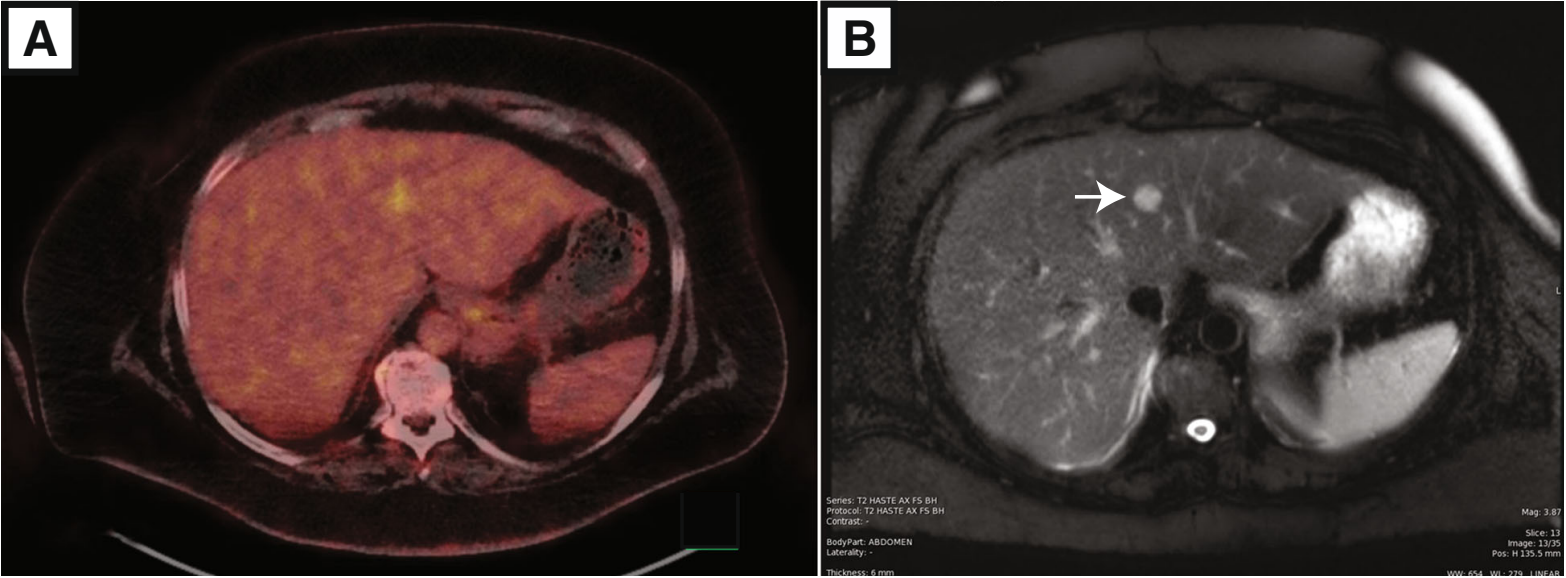
Imaging of the liver lesion. PET-CT (**a**) showed heterogenous liver uptake with a focus in the medial left lobe. T2-weighted MRI (**b**) confirmed a discrete 1.8 cm lesion suspicious for metastasis (*arrow*). This was later confirmed by biopsy

Interval surveillance of serum ACTH and 24-hour urine cortisol levels was used to monitor for recurrence. Twenty-one months after the patient’s second pituitary tumor resection, significant elevations of serum ACTH and 24-hour urine cortisol to 142 pg/mL (normal range 10–60 pg/mL) and 541 μg/day (normal range 3.5–45 μg/day), respectively, were detected (Fig. 3) despite stability of her pituitary MRI (Fig. 1e). She was thus started on mifepristone for refractory hypercortisolemia. A PET scan was performed, which demonstrated a heterogeneously hypermetabolic liver with a maximum SUV of 3.3 (Fig. 4a). MRI showed a 1.8 cm lesion suspicious for metastasis (Fig. 4b). An ultrasound-guided liver needle core biopsy was thus performed. Pathology revealed nests of tumor cells, again displaying a monotonous epithelioid appearance, abundant granular cytoplasm, and distinct nucleoli (Fig. 2c-d). Immunostains for AE1/3, synaptophysin, and ACTH diffusely highlighted the lesional cells. p53 demonstrated diffuse strong positivity, and Ki-67 highlighted an average of 10% of the tumor nuclei, with focal areas approaching 20% (Fig. 2e–h). The diagnosis of metastatic pituitary carcinoma was thus confirmed.

The patient subsequently underwent resection of the metastatic liver lesion, with surveillance testing for serum ACTH, 24-hour urine cortisol, and pituitary MRI performed every 2–3 months thereafter. Pathology on the patient’s wedge resection confirmed the findings from her needle core liver biopsy. Imaging follow up performed 6 months postoperatively, consisting of PET-CT and pituitary MRI, was negative for additional metastatic disease or local progression (Fig. 1f). Approximately 1 year after resection of the metastasis, the patient presented with subjective left periorbital edema. Significant tumor growth in the cavernous sinuses causing venous congestion was demonstrated on MRI (Fig. 1g). She was initiated on temozolomide and capecitabine oral chemotherapeutics and repeat imaging at last follow-up revealed stabilization of the intracranial lesions after 8 months of chemotherapy, 22 months after the initial diagnosis of metastatic disease (Fig. 1h). Her neurological status remains at baseline. However, she has suffered biopsy-proved recurrence of her liver metastases, which are now inoperable.

## Discussion

Numerous methods to distinguish between pituitary adenomas and lesions that will ultimately progress to carcinoma have been presented in the literature [3, 10, 20–22]. Clinically, use of this data translates into a diagnosis of APA, which per the 2004 World Health Organization (WHO) classification [23] shows a Ki-67 proliferation index >3% and reactivity for p53 protein by immunohistochemistry. These lesions, along with all adenomas showing invasive features intraoperatively or by imaging, are followed more closely in hope of detecting recurrences and early metastatic lesions.

The clinical progression of the presented case is common among reported pituitary carcinomas: invasive or otherwise atypical pituitary adenomas usually recur several times and are repeatedly resected before craniospinal or systemic metastatic lesions are discovered secondary to routine surveillance imaging, laboratory monitoring, or progressive clinical symptoms. The interval for progression to carcinoma has been reported to range from mere months to 32 years [3, 5, 11, 18]. One of the larger series of pituitary carcinomas in the literature reported a mean interval to metastasis of 6.6 years, with slightly longer time to progression (9.5 years) for ACTH-secreting tumors [3].

In the case presented herein, interval laboratory monitoring and subsequent imaging identified the metastasis 29 months (2.4 years) after the initial APA diagnosis. Because of her long-term waxing and waning hypercortisolemia of approximately 2 1/2 years’ duration, and possible acquired tolerance of increased cortisol, it is not clear however whether she was experiencing recurrence of symptoms due to growth of the metastasis. Later during her course, she was started on mifepristone, which made interpretation of her clinical symptoms relative to laboratory values even more difficult.

Despite the infrequency by which pituitary carcinomas occur, the majority of corticotroph pituitary carcinomas indeed present in association with Cushing’s disease [2], as was the case in our patient. Initially, pituitary carcinoma was suggested to have a stronger association with Nelson’s syndrome [3, 10, 11], where an already transforming corticotroph tumor is thought to get a boost toward carcinoma from the removal of excess cortisol [2], but the growing number of reports in Cushing’s disease patients continues to suggest otherwise [12–19].

The pathologic progression of APA toward pituitary carcinoma is well illustrated by our case. It is generally accepted that pituitary carcinomas gradually arise from adenomas and APAs. Here, the Ki-67 proliferation index of 4% in the initial resection progressed to up to 15% in a re-resection to up to 20% in the liver metastasis nearly 3 years later, with correspondingly more aggressive clinical features. In addition to elevated Ki-67 labeling and abnormal expression of p53 protein by immunohistochemistry, routinely used to classify adenomas as atypical [20], several other features have been suggested to help differentiate adenomas from more aggressive lesions. Higher mitotic activity [3], cellular pleomorphism and/or hyperchromasia [3], miR20a and miR-17-5p upregulation [8], increased galectin-3 expression [24], decreased p27 expression [21], increased frequency of aneuploidy [3], decreased β-catenin expression [25], decreased BCL2 expression [22], and increased topoisomerase-2α expression [26] have all been associated with APAs or their progression to pituitary carcinoma. Most of these potential markers, however, have not been incorporated into clinical diagnosis.

In this case, PET imaging demonstrated that the liver harbored the only area of concern for metastatic disease. In addition to the liver, pituitary carcinoma has been reported to spread to the cerebral cortex, cerebellum, spinal cord, leptomeninges, eyes, heart, lung, pancreas, kidney, various lymph nodes, ovary, myometrium, & bone [2, 3]. The methods of dissemination to these various sites have been separated into three distinct pathways. The first and most common is that of systemic metastasis, thought to occur by hematogenous or lymphatic dissemination [27–29]. Subarachnoid leptomeningeal spread is also commonly reported [3, 15, 30] and thought to proceed via the cerebrospinal fluid [28]. Lastly, metastases to brain parenchymal locations, such as the cortex [3, 13, 16] and cerebellum [3, 9], have been suggested to be a result of reverse venous flow [27, 28, 31].

Surgical manipulation has been implicated to play a role in metastatic spread. Lehman et al. [32] reported spinal drop metastases of an APA following multiple resections that may have been facilitated via a pressure gradient created by a lumbar drain. Another report of drop metastasis demonstrated similar histology of the primary and metastatic lesion and a Ki-67 proliferation index of <3% in the metastatic lesion, suggesting mechanical rather than biological factors contributing to disease progression [15]. Most reported cases of pituitary carcinoma have been associated with surgical intervention to a sellar lesion prior to dissemination [3, 5, 11], leading to varying opinions on the effects of surgical manipulation. However, rare cases have been reported to proceed to pituitary carcinoma without surgical manipulation [33], contributing to the individuality of cases and the subsequent difficulty in making generalizations about their course, behavior, or best treatment.

Treatment for pituitary carcinoma typically consists of surgical resection of the sellar lesion and accessible metastases, often with adjuvant radiation [24, 34, 35]. Prior to the diagnosis of carcinoma, many APAs also undergo radiation therapy for unresectable residual disease [36]. However, ionizing radiation to an APA could potentially accelerate transformation to pituitary carcinoma via radiation-induced carcinogenesis. Radiation may also facilitate metastasis by damaging blood vessels, the leptomeninges or other tissues as hypothesized in gliomas [37]. Selection bias confounds our ability to make any causative statements in this matter, since most patients receiving radiation already present with an aggressive clinical course, and the disease is rare enough that a randomized clinical trial is not possible to isolate the variable of radiation therapy on carcinoma progression. Nevertheless, if surgically feasible with low morbidity, we prefer re-resection of an APA instead of radiation, given this concern. In the present case, the fibrous nature of the tumor and its intimate association with the cavernous sinus precluded additional safe resection. We did offer the patient a craniotomy for radical surgical resection of the left cavernous sinus disease before she initiated chemotherapy, but she declined due to the potential high morbidity and non-curative nature of the procedure.

Medically, dopamine agonists are used in prolactin-secreting lesions [3]. Anecdotal reports of chemotherapy use have been reported in small series, but no definitive role for this treatment beyond possible palliation has been clearly identified [3, 9]. The use of temozolomide with or without capecitabine for pituitary carcinoma has increased in the last decade with some positive results published, but this indication is still experimental [38–41] and acquired resistance has been described [42]. New, targeted therapies may improve outcomes for ACTH-producing carcinoma in coming decades, but no such agents are yet approved for use in the United States.

Survival among pituitary carcinoma patients varies, but prognostic estimates are generally poor. Hansen et al. [33] reported 57.1 and 28.6% survival at 1 and 5 years, respectively, with others reporting median survivals at much less than a year [3, 28]. Decreased survival has been associated with increasing age, male gender, and non-Caucasian race [33]; however, small numbers of pituitary carcinoma cases in series that include survival data is a limiting factor.

## Conclusions

In summary, we present an uncommon case of ACTH-secreting pituitary carcinoma metastatic to the liver in a patient with a history of an invasive APA and Cushing’s disease. Clinical awareness of the rare possibility for invasive or atypical pituitary adenomas to metastasize is essential to appropriately monitor patients for possible early detection and treatment of pituitary carcinoma.

## Abbreviations

ACTH: Adrenocorticotropic hormone
APA: Atypical pituitary adenoma
cGY: centigray (unit of radiation)
CT: Computed tomography
FSH: Follicle stimulating hormone
H&E: Hematoxylin and eosin
IMRT: Intensity modulated radiation therapy
LH: Luteinizing hormone
MRI: Magnetic resonance imaging
PET: Positron emission tomography
SUV: Standardized uptake value
TSH: Thyroid stimulating hormone
